# Non-Hypertensive Effects of Aldosterone

**DOI:** 10.3390/ijms26020540

**Published:** 2025-01-10

**Authors:** Natalia Ekman, Ashley B. Grossman, Anna Nieckarz, Łukasz Jędrzejewski, Jacek Wolf, Dorota Dworakowska

**Affiliations:** 1 Department of Hypertension and Diabetology, Medical University of Gdańsk, 80-214 Gdańsk, Poland; n.ekman@gumed.edu.pl (N.E.); anna.nieckarz@gumed.edu.pl (A.N.); lukjedrzejewski@gumed.edu.pl (Ł.J.); jacek.wolf@gumed.edu.pl (J.W.); 2 Centre for Endocrinology, Barts and the London School of Medicine and Dentistry, Queen Mary University of London, London E1 4NS, UK; ashley.grossman@ocdem.ox.ac.uk

**Keywords:** aldosterone, the renin–angiotensin–aldosterone system (RAAS), primary aldosteronism, subclinical atherosclerosis markers, cardiovascular remodelling, obstructive sleep apnoea

## Abstract

Aldosterone, the primary adrenal mineralocorticoid hormone, as an integral part of the renin–angiotensin–aldosterone system (RAAS), is crucial in blood pressure regulation and maintaining sodium and potassium levels. It interacts with the mineralocorticoid receptor (MR) expressed in the kidney and promotes sodium and water reabsorption, thereby increasing blood pressure. However, MRs are additionally expressed in other cells, such as cardiomyocytes, the endothelium, neurons, or brown adipose tissue cells. Therefore, aldosterone (especially aldosterone excess) may have other major impacts besides simply regulating blood pressure and circulating ion balance. Recent studies have reported a non-hypertensive impact on the cardiovascular, immune, and metabolic systems, a pro-oxidant effect, and a pro-fibrotic effect. In this review, we emphasise the non-hypertension-related effects of aldosterone, including advances in our understanding of the cellular mechanisms by which aldosterone mediates its cellular effects. We also summarise potential clinical complications related to both the hypertensive and non-hypertensive effects of aldosterone.

## 1. Introduction

Aldosterone is secreted by the adrenal cortex and plays a crucial role in regulating blood pressure and maintaining proper sodium and potassium serum levels as part of the renin–angiotensin–aldosterone system (RAAS). This steroid hormone is produced in the zona glomerulosa of adrenal glands. The production of aldosterone is stimulated by angiotensin and increased potassium serum levels and interacts with the mineralocorticoid receptor (MR) to influence epithelial cells in the distal tube and collecting duct of the kidney, colonic mucosa, and sweat glands. MRs are also present in endothelial cells, vascular smooth muscle cells, cardiomyocytes, and some neurons.

In the kidneys, aldosterone causes water and sodium reabsorption, resulting in increased blood volume and blood pressure. This process is associated with potassium loss to maintain electrolyte balance. Therefore, the disruption of aldosterone production can cause specific clinical consequences.

Some conditions, such as Addison’s disease (primary adrenal insufficiency), are associated with a loss of aldosterone leading to symptoms such as fatigue and weakness, orthostatic hypotension, and salt craving. An insufficiency in aldosterone is treated with mineralocorticoid substitutions. The standard treatment is fludrocortisone, which has a ten-fold higher mineralocorticoid potency compared to aldosterone [1].

The opposite situation is an increased serum aldosterone level. The autonomous overproduction of aldosterone is known as primary aldosteronism (PA). PA is the most common cause of secondary hypertension, with a prevalence of 10% among all hypertensive patients, compared to up to 20% of patients with resistant hypertension [2]. The main manifestations of this condition are high blood pressure, an increased aldosterone serum level, and suppressed circulating renin. The treatment of PA relies on pharmacological treatment with mineralocorticoid receptor inhibitors or surgical adrenalectomy, depending on the type of disease.

These conditions are generally related to the well-known hypertensive effects of aldosterone and the loss of serum potassium; however, recent studies have increasingly reported a non-hypertensive impact on the cardiovascular, immune, and metabolic systems, plus pro-oxidant and pro-fibrotic effects. This suggests that aldosterone (especially aldosterone excess) may have a major impact on the organism, independent of the regulation of blood pressure and levels of circulating sodium and potassium ions.

## 2. Aldosterone and the RAAS

**Figure 1:**
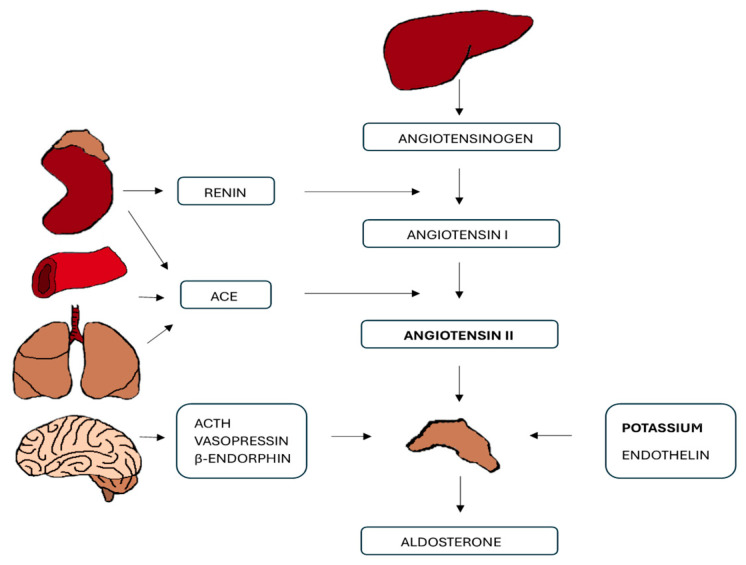
The renin–angiotensin–aldosterone system.

The renin–angiotensin–aldosterone system (RAAS) is a regulatory system that is responsible for modulating blood pressure and ion balance. The first step of this hormonal cascade is represented by angiotensinogen (Figure 1). This is a polypeptide produced in the liver, which subsequently undergoes conversion to angiotensin I (Ang I); this reaction is catalysed by renin [3]. Renin is an enzyme produced by juxtaglomerular kidney cells (JGCs), and its secretion is stimulated by renal baroreceptors and reduced sodium ions load delivered to the distal tubule of the kidney, as well as by the sympathetic nervous system-activated b1-adrenergic receptors [4]. Thus, reduced blood pressure or a reduced sodium level will cause increased production of renin, which subsequently initiates the conversion of angiotensinogen to Ang I. Ang I then is converted to angiotensin II (Ang II) by the activation of angiotensin-converting enzyme (ACE) [5]. In addition, ACE converts inactive Ang I to the active vasoconstrictor, Ang II, but also causes the deactivation of bradykinin, which is a vasodilator [6]. Thus, ACE, through its influence on the balance of vasoconstrictors and vasodilators, plays an important role in regulating blood pressure. Ang II, an active product of the reaction catalysed by ACE, is in turn responsible for the secretion of the terminal factor in the RAAS—aldosterone (Figure 1).

Ang II acts at angiotensin II type 1 receptors (AT1Rs) in zona glomerulosa cells to stimulate the production of aldosterone [7]. Aldosterone then interacts with the mineralocorticoid receptor (MR) and causes water and sodium reabsorption, resulting in increased blood volume and blood pressure.

The RAAS can be modulated therapeutically in several ways. Thus, knowledge of these mechanisms is crucial when considering anti-hypertensive treatment. Currently, medications influencing the RAAS are widely used in medicine, including ACE inhibitors, angiotensin receptor blockers, and aldosterone antagonists.

## 3. Aldosterone Biosynthesis

**Figure 2:**
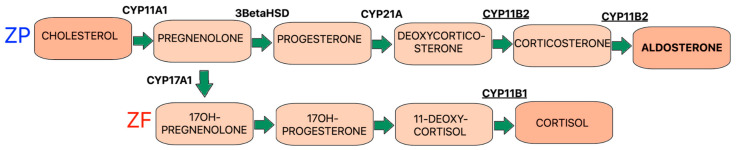
The pathway of aldosterone biosynthesis.

Aldosterone is one of the major hormones produced by the adrenal gland: the cortex of the adrenal glands is composed of three zones, the zona glomerulosa (ZG), the zona fasciculata (ZF), and the zona reticularis (ZR). The cells of each zone are responsible for the secretion of different types of adrenal hormones. Mineralocorticoids such as aldosterone are produced in the ZG, whereas ZF cells produce glucocorticoids and ZR cells mainly produce adrenal androgens (Figure 2) [8].

The precursor to these hormones is the same molecule, cholesterol, and differential enzyme expression is responsible for the final production of different adrenal hormones [9]. Enzymes such as aldosterone synthase (CYP11B2) and 11b-hydroxylase (CYP11B1) are zone-specific steroidogenic enzymes that are responsible for the final synthesis of aldosterone and cortisol, respectively. Therefore, adrenocortical cells positive for CYP11B2 and negative for cytochrome P450 17α-hydroxylase/C17-20lyas (CYP17) are typical of cells producing aldosterone, while cells producing cortisol are classified as positive for CYP11B1 and negative for CYP17 [8]. In addition, the biosynthesis of aldosterone is associated with two pathways, acute (increased synthesis after minutes) and chronic (increased synthesis after hours to days). The acute phase is controlled by rapid signalling pathways, which are related to increased concentrations of cholesterol in the mitochondria, while the chronic phase is associated with the increased expression of enzymes involved in the synthesis of aldosterone, especially CYP11B2 [10].

As noted above, the main factors regulating aldosterone secretion are Ang II and an increased serum potassium level. Ang II binds to the AT-1 receptors in ZG cells and initiate the process of aldosterone secretion through increasing calcium intracellular concentrations. Calcium is released from intracellular storage and also enters cells through the membrane calcium channel [11]. Conversely, an increased extracellular concentration of potassium causes changes in the membrane potential of ZG cells, a process controlled by membrane potassium channels. Even small changes in extracellular potassium concentrations of up to about 1 mmol/L can cause two-fold aldosterone production [11]. These changes in the membrane potential in ZG cells lead to the opening of voltage-gated calcium channels and an influx of calcium into the cell.

Generally, the secretion of aldosterone is controlled by calcium and potassium channels. Therefore, most of the mutations associated with the overproduction of aldosterone in primary aldosteronism (PA) are located on genes encoding ion channels and pumps such as KCNJ5, encoding the potassium channel, or CACNA1D, encoding part of a calcium channel [12].

## 4. Hypertensive Effect of Aldosterone

Aldosterone acts by binding to the mineralocorticoid receptor (MR), which belongs to the nuclear receptor family. Aldosterone as a lipophilic ligand diffuses into the cell cytoplasm, where it interacts with MR; the MR–aldosterone complex dimerises and moves into the nucleus, where it interacts with the promoter of genes, which induce the transcriptions of specific proteins. MRs are expressed in cells in the kidney, colon, and sweat glands, but also in cardiomyocytes, the endothelium, neurons, and brown adipose tissue cells.

The MR is not aldosterone-specific and can also interact with 11-deoxycorticosterone (a precursor of aldosterone) and cortisol. However, some tissues are known as aldosterone-selective (e.g., the kidneys and parotids), while others with the same receptor are not (e.g., the hippocampus and heart). The cells of aldosterone-selective tissues have a high expression of 11b-hydroxysteroid dehydrogenase, which protects the MR from interaction with cortisol. This enzyme converts cortisol to inactive cortisone [13], and in this manner ‘protects’ the MR from cortisol and renders it aldosterone-selective. Medications that interact with the MR are used clinically, including fludrocortisone (a strong agonist used in mineralocorticoid replacement therapy) and spironolactone or eplerenone (antagonists of MR used in hypertension treatment, especially in PA).

In the distal tubules and collecting ducts of the kidney, the activation of MRs initiates genetic transcription and translation processes, finally leading to the synthesis of a specific protein—the epithelial sodium channel (ENaC). The ENaC is a sodium channel mainly located in the apical membrane of nephron epithelial cells. Aldosterone increases the number and the activity of ENaCs and thus stimulates transepithelial sodium transport from urine [14]. This sodium reabsorption creates a hyperosmolar environment, causing the parallel influx of water from the urine into the blood. As a result, the blood volume increases, leading to an elevation in blood pressure. Additionally, this process is accompanied by potassium excretion to achieve electrolyte balance. In addition, aldosterone can directly induce vasocontraction, which also results in increased blood pressure.

Physiologically, all these mechanisms are used to regulate and control blood pressure and electrolyte serum levels within defined homeostatic limits. However, changes in aldosterone production, such as overproduction, can trigger pathological processes resulting in hypertension.

## 5. Primary Aldosteronism

Primary aldosteronism (PA) is associated with the excessive and autonomous production of aldosterone. PA is the most common cause of secondary hypertension. The prevalence of PA is now considered to be around 10% of all hypertensive patients and increases with the severity of hypertension. In patients with resistant hypertension, 20% of cases may actually be due to PA. The principal aetiology of PA includes bilateral idiopathic hypertrophy (BIH) and aldosterone-producing adenomas (APAs), while less frequent causes include unilateral hyperplasia, familial hyperaldosteronism (FH) types I-IV, aldosterone-producing carcinoma, and ectopic aldosterone synthesis. The three characteristic signs of PA are an increased aldosterone serum level, a reduced renin serum level, and hypertension. Hypokalaemia has been considered a major feature of PA, but recent studies have shown that hypokalaemia is present only in 28% of PA patients [15].

The main symptom of PA is hypertension, which is often resistant and difficult to treat. PA patients can also complain of headaches, visual problems, fatigue, and symptoms associated with hypokalaemia such as muscle cramps and weakness or numbness. The diagnosis of PA is complex and consists of three main steps: the evaluation of the aldosterone–renin ratio, usually a confirmatory test, and a subtyping test. The subtyping tests includes CT scanning and adrenal vein sampling (AVS). Distinguishing between the unilateral and bilateral forms of the disease is crucial because it will lead to the implementation of the most appropriate treatment, which differs according to the cause. Unilateral PA can be treated surgically (adrenalectomy), whereas the bilateral form of PA should be treated pharmacologically with MRA such as spironolactone or eplerenone.

Patients with PA have increased cardiovascular risk secondary to their hypertension. However, recent studies have reported that patients with PA have a higher rate of cardiovascular events than patients with essential hypertension (EH) with comparable blood pressure [16,17]. PA patients more frequently have significant left-ventricular hypertrophy and increased aortic stiffness compared to age-, sex- and degree of BP-macheted patients with EH [18]. This specific effect seen in PA is probably directly related to the increased aldosterone level and its interaction with MRs present in different tissues such as cardiomyocytes or the endothelium and independent of the hypertensive effect.

## 6. Impact of Aldosterone on the Cardiovascular System

Aldosterone is considered a mediator of cardiovascular damage [19]. The overproduction of aldosterone per se can cause pathological changes in the cardiovascular system. Aldosterone acting on the MR can damage the endothelium of vessels, stimulate inflammation, and subsequently enhance the development of atherosclerotic plaques and arterial stiffness [19]. In addition, it can lead to myocardial insufficiency through the development of myocardial hypertrophy as a result of the enhancement of inflammation and fibrosis. According to data from the *German Conn Registry*, cardiovascular mortality is the main cause of death among PA patients (50% versus 34% in matched hypertensive controls) [20].

**Figure 3:**
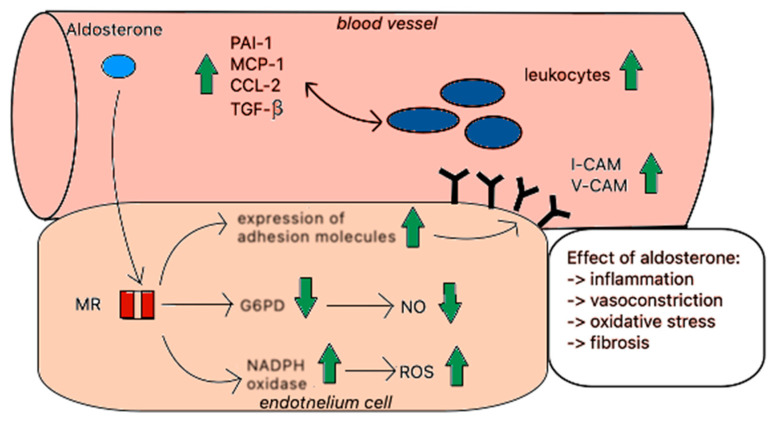
The effect of aldosterone in the endothelium.

Aldosterone damages endothelial cells in different ways (Figure 3) but is mainly associated with the impairment of nitrogen oxide (NO) synthesis: NO is a signalling molecule that plays a crucial role in regulating vascular tone and blood flow; it stimulates vasodilatation and thus reduces vascular resistance, lowers blood pressure, and increases blood flow. Recent studies have shown that aldosterone inhibits endothelial NO synthase (eNOS) activity in vitro and subsequently reduces the production of NO [21]. In addition, aldosterone increases the amount of reactive oxygen species and reduces the bioavailability of NO by inhibiting glucose-6-phosphate dehydrogenase (G6PD) in endothelial cells [22]. This impairment of NO production and the consequent reduction in endothelial NO bioavailability can enhance other pathological changes such as the development of atherosclerotic plaque or arterial stiffness in PA patients. The early investigation of these changes could be crucial in the primary prevention of cardiovascular disease in PA patients.

Furthermore, aldosterone induces the production of proinflammatory factors such as monocyte chemoattractant protein-1 (MCP-1), C-C motif chemokine ligand 2 (CCL2), chemokine C-X3-C motif ligand 1 (CX3CL1), and C-C motif ligand 5 (CCL5), causing the influx of leukocytes, mainly monocytes/macrophages [23]. The activation of MRs in monocytes/macrophages increases the expression of proinflammatory cytokines and oxidative markers that represent a specific component of NADPH oxidase—p22phox and plasma activator inhibitor-1 (PAI-1) [24]. Aldosterone, via MRs present in the endothelium, increases the expression of adhesion molecules, including the intercellular adhesion molecule-1 (ICAM-1) and the vascular cell adhesion molecule-1 (VCAM-1). The increasing density of VCAM-1 and ICAM-1 promotes the adhesion of leukocytes to endothelial cells and infiltration into intercellular spaces [24]. All these processes are associated with inflammation and enhance atherosclerosis. One study investigated the influence of aldosterone on atherosclerosis in mice with apolipoprotein E (Apo-E) deficiency. Researchers have shown that eplerenone reduces oxidative stress and inflammation and reduces the subsequent atherosclerosis lesions in apoE-deficient mice fed a high-cholesterol diet. These results suggest that aldosterone may play a crucial role in the atherosclerosis process and eplerenone may prevent these changes [25]. Another study demonstrated that macrophages isolated from aldosterone-treated mice had an enhanced ability to oxidise low-density lipoprotein (oxLDL) and increase superoxide anion production, whereas MR blockage reduced these effects [26]. These results support a role for MR signalling in endothelial cells in the initial stages of atherosclerosis.

Aldosterone also contributes to the impairment of cardiac function. It stimulates cardiomyocyte hypertrophy and myocardial fibrosis by directly increasing fibroblasts [23]. Normal cardiac tissue contains MRs. Cardiac MRs are located in cardiomyocytes, cardiac fibroblasts, and endothelial cells of the coronary arteries. Therefore, the effect of aldosterone on the heart is multifaced and complex. Aldosterone induces the expression of cyclooxygenase 2 (COX-2), MCP-1, or osteopontine in the myocardium. It also increases ICAM-1 and VCAM-1 on the surface of the coronary artery endothelium. The study “aldosterone induces a vascular inflammatory phenotype in the rat heart” demonstrated that aldosterone may cause leucocyte infiltration (mainly macrophages) and injury in coronary arteries with ischemic and necrotic damage to the surrounding myocardium. Researchers also observed the upregulation of proinflammatory molecules (ostepotin, MCP-1 and COX-2) in rat hearts in response to aldosterone [27]. In addition, the study showed that MR blockage reduces the expression of these proinflammatory molecules and inhibits subsequent cardiac damage, which indicates the role of aldosterone in myocardial injury. This study lasted only 4 weeks, and there was no evidence of significant myocardial fibrosis [27]. However, aldosterone is also associated with cardiac remodelling and fibrosis. The study “Deletion of Mineralocorticoid Receptors from Macrophages Protects Against Deoxycorticosterone/Salt-Induced Cardiac Fibrosis and Increased Blood Pressure” showed that MR signalling in macrophages is essential for triggering fibrosis in the heart. Macrophages, via the secretion of pro-fibrotic molecules such as Ang II, transforming growth factor β (TGF-β), and cytokines, play a crucial role in initiating fibroblast differentiation into myofibroblasts, important collagen-producing cells [28]. Aldosterone also increases the expression of AT-1R in the ventricle myocardium and thus enhances the pro-fibrotic role of Ang II in the heart [PMID: 10205234]. In addition, aldosterone promotes the production of endothelin-1, which is associated with collagen-1 secretion [29].

All the processes described above cause structural the remodelling of the heart and consequently can lead to cardiac insufficiency. Furthermore, aldosterone can negatively impact coronary arteries and increase the risk of myocardial infarction. Rocha and colleagues reported that aldosterone caused severe hypertension and vascular inflammation in the coronary arteries in uninephrectomized rats [27].

Numerous studies both in vivo and in vitro have demonstrated the impact of aldosterone on vascular dysfunction and myocardial remodelling [27,30,31,32]. Thus, the aldosterone–MR signalling pathway is considered a major factor in the pathophysiology of cardiovascular (CV) disease, and it could be possible to use this association in primary and secondary CV disease prevention [33]. Indeed, several clinical studies have demonstrated the correlation between aldosterone–MR activation and CV risk, while others have shown the influence of aldosterone in the development of CV disease [34].

The *Randomized Aldactone Evaluation Study* (RALES) evaluated the effect of spironolactone in patients with systolic hypertension and an LVEF (left-ventricle ejection fraction) of or below 35% and concluded that this agent reduced the risk of mortality and hospitalisation for heart failure [34]. In another study—EPHESUS (the *Eplerenone Post-Acute Myocardial Infraction Heart Failure Efficacy and Survival Study*)—the authors investigated the influence of eplerenone on CV mortality and morbidity in patients with acute heart failure, comprising patients with heart failure after myocardial infarction with an LVEF < 40%. The study was randomised, with the patients receiving eplerenone or a placebo. EPHESUS showed that the use of eplerenone in post-myocardial infarction patients with left-ventricular dysfunction and heart failure led to a significant reduction in CV mortality and morbidity [34].

## 7. Aldosterone and Subclinical Atherosclerosis Markers

**Figure 4:**
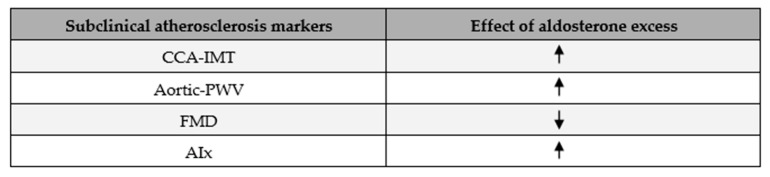
The correlation between aldosterone excess and subclinical atherosclerosis markers.

Subclinical atherosclerosis is a well-known marker of CV disease, and clearly, any involvement of aldosterone in its genesis is clinically important. Many studies have shown that aldosterone excess enhances the development of atherosclerosis, and this is associated with increased total CV risk for PA patients [17,35,36,37]. Early investigation into such subclinical changes can be crucial in the primary prevention of CV diseases in patients with PA. In terms of the evaluation of subclinical atherosclerosis, useful parameters might include the common carotid artery intima-media thickness (CCA-IMT), prevalence of carotid plaques, flow-mediated dilation (FMD), nitrate-mediated dilation (NMD), pulse-wave velocity (PWV), augmentation index (AIx), and ankle–brachial index (ABI) (Figure 4).

The CCA-IMT is defined as a measurement of the thickness of the inner and middle part of the carotid wall and is assessed using non-invasive techniques such as ultrasonography of the carotid arteries, which can also assess the prevalence of carotid plaques [38]. No special patient preparation for this examination is necessary. The first step is the visualisation of the common carotid artery (CCA) using a suitable linear array transducer (usually 7–12 MHz) to obtain clear B-mode ultrasound images of the CCA. The IMT is measured from the leading edge of the first echogenic line (lumen–intima interface) to the leading edge of the second echogenic line (media–adventitia interface). The measurement of CCA-IMT is typically assessed in the distal wall of the CCA, located approximately 1 cm before the bifurcation of the artery [39]. In this segment of the CCA, multiple measurements of IMT are taken, and an average of these values is computed. This location generally provides clear images and more reliable measurements. An increased CCA-IMT is associated with higher CV risk [40].

FMD and NMD are all non-invasive ultrasonographic methods and are used to assess the function of the arterial endothelium by the measurement of the changing diameter of the artery.

Another parameter used in clinical practice to assess subclinical atherosclerosis is the ankle–brachial index (ABI). The ABI is a simple, non-invasive method of evaluating the risk of peripheral arterial disease (PAD) [41]. PAD is associated with lower extremity arterial perfusion, which is generally caused by atherosclerotic plaques [42]. ABI is evaluated by dividing the systolic blood pressure at the ankle by the systolic blood pressure in the arm. The equipment needed for this examination comprises a pneumatic cuff and a continuous-wave Doppler probe. Blood pressure cuffs are attached to the arm and above the ankle. The Doppler device is used to listen to the arterial blood flow. ABI can also be evaluated by using automatic ABI measurement devices [43]. Normal ABI values are in the range 1.0–1.4, whereas values below 1.0 are associated with an increased risk of PAD. Values greater than 1.4 are associated with the calcification of vessels, often seen in elderly people and patients with chronic kidney disease or diabetes [44].

Many patients with PAD are asymptomatic, but they have an increased CV risk because PAD is a marker of systemic atherosclerosis. Therefore, the assessment of ABI could be important in primary CV prevention [42].

Another aspect of disease potential associated with aldosterone excess is arterial stiffness. Arterial stiffness refers to reduced arterial distensibility. It is connected to smooth muscle cell contraction (active stiffness) and the arrangement of structural components within the arterial wall, such as elastin or collagen (passive stiffness). Elevated arterial stiffness is considered as an independent predictor of cardiovascular events and mortality [45]. It is associated with hypertension, coronary artery disease, or stroke.

Aldosterone, through its interaction with MRs located in vascular smooth muscle cells (VSMCs), enhances active arterial stiffness. It causes an increase in vascular myogenic tone and agonist-dependent contraction [46]. In addition, aldosterone also enhances passive arterial stiffness by stimulating the arrangement of the structural components of the arterial wall. Aldosterone causes extracellular matrix remodelling associated with the accumulation of grown factors and stimulates altered elastin and collagen, increased inflammation, VSMC hypertrophy, and fibrosis [47]. These processes consequently lead to stiffness and also the calcification of the arterial wall [48].

Non-invasive methods of evaluating arterial stiffness include PWV and AIx. Of these two methods, PWV, as a direct measurement, is more precise [49,50]. PWV reflects the velocity of the pulse wave moving between proximal and distal artery sites. The measurement of PWV is also non-invasive but requires specialised equipment. PWV devices use tonometry or oscillometric methods to detect pulse waves [51], with tonometric carotid-femoral PWV (cfPWV) measurements currently considered the gold standard for estimating arterial stiffness [51]. In carotid–femoral PWV (cfPWV), one sensor is located at the carotid artery on the neck and another at the femoral artery on the groin. The distance between these two points is measured, and then PWV devices calculate the transmission time of the pulse wave. The velocity is the ratio of the distance between the sensors to the transit time of the pulse wave [52]. Increased values of PWV indicated arterial stiffness. Increased cfPWV is a direct measurement that reflects aortic stiffness and is associated with elevated total CV risk [53].

Ambrosino et al. analysed a number of the parameters mentioned above in participants with EH, with PA, and without hypertension from 12 case–control studies in total and reported that those who suffered from PA had a higher CCA-IMT and aortic PWV than EH and normotensive groups. Furthermore, Aix, as well as AIx normalised to a heart rate of 75 beats per minute (AIx@75), were higher, and FMD was lower, in patients with PA compared to normotensives [18]. In general, these data demonstrate that aldosterone itself enhances the progression of subclinical atherosclerosis.

## 8. Aldosterone and Atrial Fibrillation

Numerous studies have demonstrated that PA patients have a higher risk for the development of atrial fibrillation (AF) than patients with essential hypertension with comparable blood pressure [54,55]. Milliez and coworkers reported that PA was associated with a 12.1-fold higher risk of AF compared to essential hypertension [16]. A recent meta-analysis of 31 studies including 3838 patients with PA reported that the risk of AF was 3.5-fold higher [56]. Thus, aldosterone excess significantly affects the risk of AF.

AF is the most common type of cardiac arrhythmia. The occurrence of AF depends on three main mechanisms: automaticity (impulse is spontaneously generated by cardiac cells), triggered activity (activity is triggered by additional impulses such as after-depolarisation), and re-entry (impulse circulates repeatedly within a circuit) [57]. Knowledge regarding the mechanism behind AF is crucial and can be useful in the primary and secondary prevention of AF.

**Figure 5:**
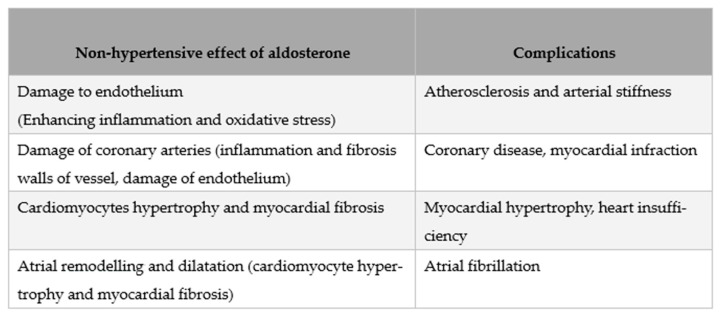
Non-hypertensive effect of aldosterone overproduction and possible consequences.

Studies have shown that aldosterone, independent of any anti-hypertensive effect, influences the development of AF in various ways. Aldosterone, through the activation of the MR, causes myocardial hypertrophy and myofibroblast stimulation, which leads, as a consequence, to atrial remodelling and dilatation (Figure 5) [19,58,59]. These structural changes in the atrium can double the time required for the spontaneous conversion of AF into sinus rhythm, promoting long-lasting AF stabilisation [60]. Moreover, aldosterone may also influence the electrophysiological properties of cardiomyocytes, increasing the cytosolic calcium concentration through the stimulation of different types of calcium channels [61]. The increased activity of calcium channels causes calcium influx in the cytoplasm and releases calcium from the sarcoplasmic reticulum following ryanodine receptor stimulation [62]. The consequent calcium overload in cardiomyocytes facilitates the onset of after-depolarisation, which can trigger AF [19]. Additionally, research investigating the relationship between PA and the prevalence of AF has demonstrated that the infusion of aldosterone in rat models causes electrocardiography (ECG) changes. Researchers have observed a significantly prolonged P-wave duration, PQ interval, and QRS duration in rats after aldosterone infusion compared with controls [55]. These ECG changes, particularly PQ prolongation, may facilitate the re-entry mechanism and thus increase AF risk [55].

The prevalence of AF in PA patients is significantly increased, although the influence of aldosterone on the development of AF is complex. Therefore, some researchers have investigated the role of aldosterone in primary and secondary AF prevention. A meta-analysis of randomised controlled trials and observational studies has shown that MR antagonists significantly reduce new-onset AF and recurrent AF [63]. Using a database of the EMPHASIS-HF (*Eplerenone in Mild Patients Hospitalization And Survival Study in Heart Failure*) study, researchers analysed the incidence of new AF or atrial flutter (AFF). In the conclusion of this investigation, it was reported that eplerenone reduced the incidences of new-onset AFF in patients with systolic heart failure and mild symptoms [64]. Moreover, according to the *European Society of Cardiology Guidelines* in 2020, MR antagonists are considered non-anti-arrhythmic drugs with anti-arrhythmic properties (upstream therapy) [65].

## 9. Aldosterone and the Neurological System

Aldosterone can influence the central nervous system (CNS) indirectly through increasing blood pressure (the hypertensive effect of aldosterone) or via its impact on the CV system (the impairment of vessels or an increasing risk of AF, as described in previous sections), which consequently can lead to neurological diseases such as ischaemic stroke or intracranial haemorrhage [66]. In a systemic meta-analysis, researchers have shown that PA patients have a higher risk of stroke compared with patients with essential hypertension. This suggests that the risk of stroke is also increased independently of the hypertensive effect of aldosterone [56].

In their research, Dinh et al. proved that aldosterone increased the production of superoxide and upregulated the mRNA expression of cytokines, including chemokine (C–C motif) ligand 7 (CCL7), chemokine (C–C motif) ligand 8 (CCL8), and interleukin-1 beta (IL-1β), through the activation of mineralocorticoid receptors in endothelial cells within the brain. These processes contribute to oxidative stress and inflammation in brain tissue. Notably, the effects of aldosterone described were independent of its blood pressure-elevating actions [67].

Moreover, recent studies have demonstrated that aldosterone can also influence CNS directly through MRs present in the brain.

MRs are extensively present throughout the central nervous system (CNS). The expression of MRs has been shown to be present in brain regions such as the hippocampus, amygdala, and prefrontal cortex [68]. The MR is unique compared with other steroid receptors insofar as it interacts with two physiological ligands, aldosterone and cortisol [69]. In the epithelial tissue, aldosterone binds to the MR in the presence of the 11b-hydroxysteroid dehydrogenase type 2 (HSD2), which converts active cortisol to cortisone [70]. In the CNS, however, cortisol is the primary ligand for the MR [71]. Aldosterone shows surprisingly poor penetration of the blood–brain barrier, which limits its action in the CNS [72]. Furthermore, only brain regions with the co-expression of HSD2 and the MR are likely to be sensitive to aldosterone, as cortisol is present in much higher concentrations compared to aldosterone [73].

Nevertheless, some neurons within the CNS show an unusual sensitivity to aldosterone. Researchers investigating MR immunoreactivity across the entire brain identified distinct clusters of neurons within the nucleus of the solitary tract (NTS) characterised by dense nuclear and perinuclear MR localization [74]. Notably, these neurons also express HSD2, an enzyme essential in aldosterone sensitivity. This population of HSD2 neurons constitutes a relatively small group within the NTS [74].

The NTS, located in the medulla oblongata and forming the brainstem, is associated with processing and integrating sensory information related to cardiovascular, respiratory, and gastrointestinal regulation [75]. Despite this, the specific effects of aldosterone on this small group of HSD neurons within the NTS remain unclear. In one study, aldosterone infusion into the fourth ventricle caused increased sodium intake and a reduction in baroreflex sensitivity, without any change in blood pressure or renal sodium excretion. The results of this study suggest that the role of aldosterone in CNS is involved in the rapid regulation of sodium intake [76,77].

In summary, HSD2 neurons are the only cells in the CNS for which there is conclusive evidence of physiological aldosterone sensitivity, but the role of aldosterone action in the CNS is still unclear [78]. Furthermore, the presence and localization of aldosterone-sensitive HSD2 neurons have been confirmed in animal studies. More research on this topic is needed.

## 10. Aldosterone and Obstructive Sleep Apnoea

Obstructive sleep apnoea (OSA) is a well-established disorder affecting a significant number of patients and is characterised by recurrent episodes of apnoea and hypopnoea during sleep, as a result of a complete or partial block in airflow [79]. This condition leads to several significant pathophysiological changes, including the activation of the renin–angiotensin–aldosterone system (RAAS) and the overproduction of aldosterone, which largely contribute to the development of arterial hypertension and its consequences. Elevated aldosterone levels can exacerbate the severity of OSA by promoting upper airway edoema and increasing the likelihood of airway collapse during sleep. Furthermore, the relationship between aldosterone and OSA is bidirectional: OSA can lead to increased aldosterone production through mechanisms such as hypoxia and sympathetic activation, creating a vicious cycle that worsens both conditions [80].

**Figure 6:**
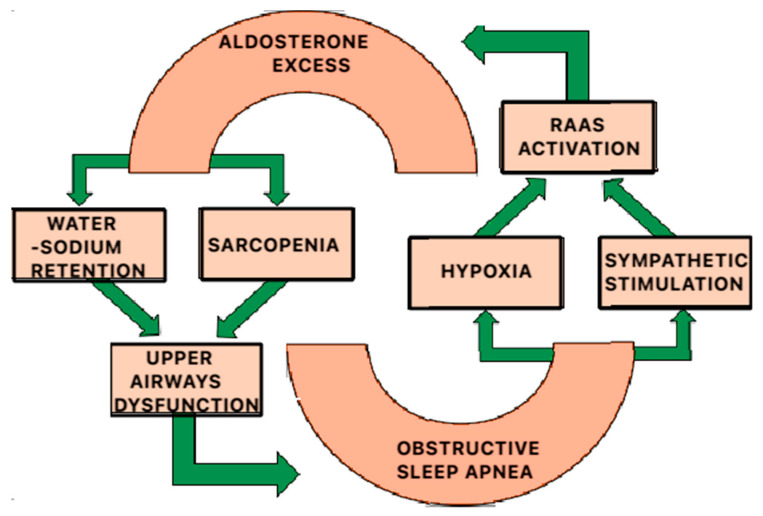
The correlation of aldosterone excess and OSA.

The bidirectional mechanism by which the excess of aldosterone influences the occurrence and exacerbation of obstructive sleep apnoea (OSA) and vice versa is still not well understood, and theories are based on established knowledge regarding the pathophysiological changes caused by hyperaldosteronism [80,81,82,83]. An increased aldosterone level can induce sodium retention and associated fluid overload in the body, leading to soft tissue oedema in the neck, throat, and larynx, particularly in the supine position, causing obstruction to the upper airways and thereby exacerbating the symptoms of obstructive sleep apnoea (Figure 6) [80,84]. On the other hand, during sleep, patients with obstructive sleep apnoea experience intermittent hypoxemia, which, in itself and through the stimulation of the sympathetic nervous system, activates the renin–angiotensin–aldosterone system (RAAS), leading to an excess of aldosterone in the body (Figure 6) [81,84]. Additionally, chronic aldosterone excess may contribute to sarcopenia, including in the skeletal muscles responsible for upper airway dilation, significantly impairing their function [80].

Currently, numerous studies are being conducted on the co-occurrence of OSA and primary aldosteronism (PA). These studies have been performed in various ethnic groups with hypertension, especially resistant hypertension, considering the severity of OSA and the advancement of other comorbidities, including obesity [80,85,86]. It has been shown that OSA occurs more frequently in patients with resistant hypertension with PA than in its absence [87]. Other studies have demonstrated a higher prevalence of PA among patients with OSA compared to those with essential hypertension [80,88]. Some results suggest that moderate-to-severe OSA with resistant hypertension might be useful in predicting the occurrence of PA [89]. The current analysis also shows a bidirectional interaction between OSA and PA [90]. It seems that hyperaldosteronism exacerbates the course of OSA, while RAAS activation caused by OSA contributes to poorer control of PA [80]. The treatment of PA, both pharmacological (aldosterone antagonist) and surgical (adrenalectomy), significantly reduces the symptoms OSA (significant improvement in the Apnoea-Hypopnea Index (AHI)) [91] as well as the risk of OSA (assessed using the Berlin questionnaire) [92,93]. Blocking aldosterone overproduction thus appears to be an effective supportive therapy in patients with both OSA and hypertension associated with PA [80,94,95]. There is an ongoing discussion regarding the benefits of screening for PA in all hypertensive patients with OSA and the routine use of aldosterone antagonists in this group [81].

It is reasonable to conduct further research to thoroughly analyse the links between PA and OSA, including the assessment of pathophysiological, genetic, and phenotypic associations of both conditions and the optimisation of management and treatment in this patient group [96].

## 11. Conclusions

Aldosterone is a multifaceted hormone with extensive effects on blood pressure regulation, cardiovascular health, neurological function, and conditions in other organs, like the lungs or kidneys. Its involvement in hypertension, cardiovascular remodelling, atrial fibrillation, and obstructive sleep apnoea underscores the importance of the early detection and targeted treatment of aldosterone-related disorders to prevent long-term adverse health outcomes.
